# Epigenomics and Non-Coding RNAs in Soybean Adaptation to Abiotic Stresses

**DOI:** 10.3390/ijms262311527

**Published:** 2025-11-27

**Authors:** Kinga Moskal, Bartosz Tomaszewski, Maja Boczkowska

**Affiliations:** Plant Breeding and Acclimatization Institute—National Research Institute, Radzików, 05-870 Błonie, Poland; k.moskal@ihar.edu.pl

**Keywords:** epigenomics, soybean, non-coding RNA, abiotic stress

## Abstract

This review presents soybean responses to drought, heat, and salinity within a signal–transcript–chromatin framework. In this framework, calcium/reactive oxygen species and abscisic acid cues converge on abscisic acid-responsive element binding factor (ABF/AREB), dehydration-responsive element binding protein (DREB), NAC, and heat shock factor (HSF) families. These processes are modulated by locus-specific chromatin and non-coding RNA layers. Base-resolved methylomes reveal a high level of CG methylation in the gene body, strong CHG methylation in heterochromatin, and dynamic CHH ‘islands’ at the borders of transposable elements. CHH methylation increases over that of transposable elements during seed development, and *GmDMEa* editing is associated with seed size. Chromatin studies in soybean and model species implicate the reconfiguration of salt-responsive histone H3 lysine 27 trimethylation (H3K27me3) in *G. max* and heat-linked H2A.Z dynamics at thermoresponsive promoters characterized in *Arabidopsis* and other plants, suggesting that a conserved chromatin layer likely operates in soybean. miR169–*NF*-*YA*, miR398–*Cu*/*Zn Superoxide Dismutases*(*CSD*)/*copper chaperone of CSD*(*CCS*), miR393–*transporter inhibitor response1*/*auxin signaling F*-*box* (*TIR1*/*AFB*), and miR396–*growth regulating factors* (*GRF*) operate across leaves, roots, and nodules. Overexpression of lncRNA77580 enhances drought tolerance, but with context-dependent trade-offs under salinity. Single-nucleus and spatial atlases anchor these circuits in cell types and microenvironments relevant to stress and symbiosis. We present translational routes, sentinel epimarkers (bisulfite amplicons, CUT&Tag), haplotype-by-epigenotype prediction, and precise cis-regulatory editing to accelerate marker development, genomic prediction and the breeding of resilient soybean varieties with stable yields.

## 1. Introduction

The soybean (*Glycine max* (L.) Merr.) is an annual legume native to East Asia. Currently, the most important legume in the world serves as a staple ingredient in food, animal feed, and many industrial products [1]. According to FAOSTAT statistics, the area under cultivation has increased almost 6-fold over the past 60 years, whereas global soybean production has increased almost 14-fold [2]. Market analyses estimate the sector’s current value at approximately USD 155 billion, with further growth expected by 2031 [3].

Soybean is widely cultivated for their edible seeds, which are highly valued for their protein and oil contents. It is consumed in various forms, including fermented products (i.e., soy sauce and tempeh), unfermented products (i.e., tofu and soy milk), and as a significant source of protein and oil in processed foods and livestock feed. Its nutritional value originates from its complete protein profile, significant omega-3 fatty acid content, isoflavones, and essential minerals and vitamins [4]. In addition to their nutritional and industrial value, soybean supports sustainable agriculture by fixing atmospheric nitrogen, thereby increasing soil fertility and reducing the need for synthetic nitrogen fertilizers [5].

Soybean is remarkably adaptable and cultivated in a variety of geographical regions. However, progressive climate change, including intensified drought, extreme heat, increased soil salinity, and more frequent and severe weather extremes, puts soybeans at risk. These factors induce significant genotype–environment interactions, which affect both yield stability and nutritional quality [6]. A lack of water during the flowering and pod-filling stages can have a significant effect on the number and weight of seeds produced. This results in a decrease in total yield per unit area and an adverse effect on protein and fat contents [7,8]. Heat stress can accelerate plant development by shortening various developmental stages, affecting the height and quality of the yield [9]. An increase in salinity disrupts ionic balance and osmotic regulation, reducing root growth and nutrient transport and further compromising yield and quality [10,11]. In practice, these stress factors often occur simultaneously. Recent studies indicate that the combination of drought and heat can have an effect on plant physiology, yield, height, and quality that is greater than the sum of its effects on the abovementioned characteristics [12].

In recent years, significant progress has been made in understanding the regulatory systems underlying soybean adaptation to abiotic stress. Epigenetic pathways, such as DNA methylation and histone modification, as well as the entire spectrum of non-coding RNAs (especially microRNAs and long non-coding RNAs), have been proven to modulate gene transcription and phenotypic plasticity under environmental stress [13,14]. Comprehensive multiomic strategies, including genomics, transcriptomics, epigenomics, and phenomics, have enabled the analysis of pathways linking environmental influences to genotype and phenotype [15,16,17]. Pangenomic analyses and single-cell atlases have revealed the full range of molecular responses to stress and their impact on seed yield, fat content, and protein composition [18,19]. Databases such as SoyMD (https://ngdc.cncb.ac.cn/databasecommons/database/id/9112 (accessed on 23 November 2025)) and SoyOD (https://bis.zju.edu.cn/soyod/home/ (accessed on 23 November 2025)) integrate these diverse datasets, which facilitates the identification of quantitative trait loci (QTLs), regulatory variants, and associated epigenetic marks. These can be used in modern breeding programs [16,17,20]. Marker-assisted selection, genomic prediction, and targeted genome editing are used in breeding programs to optimize key traits for stress tolerance, increased yield, and improved product quality [16,21]. The development of high-yielding soybean varieties adapted to climate change is promising, with the latest advances in epigenomics and multiomics research, along with advanced molecular breeding tools, offering a promising pathway. These innovations are essential for ensuring the global supply of soybeans and their derived products amid growing climatic, agronomic, and demographic challenges.

This review shows that abiotic signals (drought, heat, and salinity) propagate from the initial Ca^2+^/ROS and hormonal cues to transcriptomic reprogramming and locus-specific chromatin control. The objective is to present this information as an integrated signal–transcript–chromatin framework. Specifically, we do three things. First, we inventory the epigenomic and non-coding RNA layers relevant to soybean. These include DNA methylation with TE-proximal CHH “islands,” histone marks, accessibility, and non-coding RNA programs. Second, we relate these layers to stress-responsive transcription factor networks (ABF/AREB, DREB, NAC, HSF) and their organ contexts. Third, we translate convergent nodes into breeding-oriented workflows. These include sentinel epimarker panels (bisulfite amplicons, CUT&Tag), haplotype-by-epigenotype prediction, and precise cis-regulatory editing for yield stability under combined stresses.

Throughout this review, we explicitly distinguish three levels of evidence. First, soybean-specific datasets, including functional genetics and multiomics (marked by *Gm* gene symbols or gma-miRNA nomenclature), provide direct support for mechanisms in *Glycine max*. Second, comparative evidence from other crops and legumes is used when soybean data are limited, but legume conservation is likely. Third, mechanistic insights from model species such as *Arabidopsis thaliana* and rice are treated as conserved regulatory logic that we propose as working hypotheses for soybean. Whenever we extrapolate from these models, we state this explicitly.

## 2. Signal–Transcript–Chromatin: An Integrated Model of Soybean Responses to Drought, Heat, and Salinity

Abiotic stress responses in soybean plants emerge from a coordinated flow of information linking rapid perception and signaling to transcriptional reprogramming and chromatin-level control. Drought, heat, and salinity each initiate partially overlapping cascades—abscisic acid (ABA)-dependent and -independent modules under water deficit, heat shock factor–chaperone circuits during thermal stress, and ion homeostasis pathways under salinity—that converge on nodal transcription factors and small RNA hubs. These RNA-guided layers interact with DNA methylation, histone modifications, and changes in chromatin accessibility, calibrating the amplitude, timing, and tissue specificity of gene expression. It creates a dynamic regulatory architecture that allows for short-term priming and, in certain situations, persistence across stress–recovery cycles. As field performance is influenced by interactions between genotype and environment and frequent combinations of stress, a unified signal–transcript–chromatin framework is crucial for interpreting multiomics data, identifying transferable control nodes and prioritizing targets for breeding. Here, these drought, heat, and salinity levels are synthesized, and then, shared versus stress-specific nodes and hormone crosstalk with translational implications for marker development, genomic prediction, and precise cis-regulatory editing are delineated.

### 2.1. Drought: ABA Signaling, Transcription, and Epigenetic Regulation

**Figure 1:**
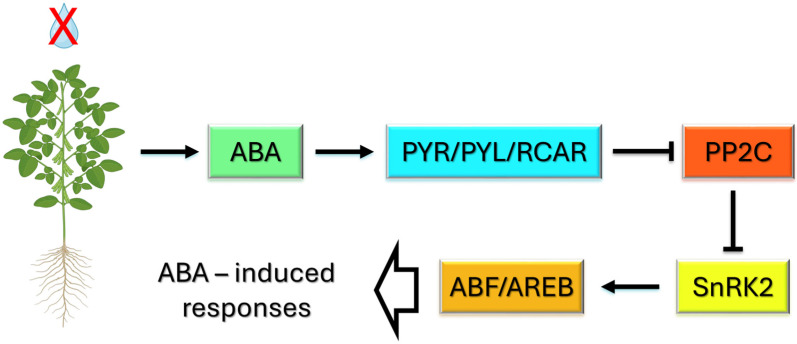
Canonical abscisic acid (ABA) signaling under water deficit conditions. Drought/low water potential promotes the accumulation of ABA, which binds to PYR/PYL/RCAR receptors (pyrabactin resistance/PYR1-like/regulatory components of ABA receptors). The ABA–receptor complex inhibits clade-A PP2Cs (type-2C protein phosphatases), thereby releasing SnRK2 kinases (SNF1-related protein kinases 2) from repression and enabling their activation. Activated SnRK2s phosphorylate the bZIP transcription factors ABF/AREB (ABRE-binding factors/ABA-responsive element-binding proteins), which drive ABA-induced gene expression and physiological responses (e.g., stomatal closure, and osmotic adjustment). The arrows indicate activation, and the blunt lines indicate inhibition; the scheme is simplified for clarity.

Drought stress in soybean plants results in a rapid and substantial increase in the ABA concentration, which is central to orchestrating the adaptive response of the plant. An increase in ABA is primarily achieved through enhanced biosynthesis in root tissues upon water deficit [22]. ABA subsequently acts as a key signaling molecule, activating a well-conserved transduction cascade. The PYR/PYL/RCAR receptor family has been demonstrated to interact with and inhibit clade A protein phosphatases type 2C (PP2Cs) upon hormone binding (Figure 1) [23]. This inhibition results in the release of sucrose nonfermenting 1-related protein kinase 2 (SnRK2) proteins, particularly subclass III SnRK2s. This process enables them to phosphorylate downstream targets. Recent studies have indicated that the activation of subclass III SnRK2s by autophosphorylation and Raf-like kinases (specifically B2- and B3-type RAFs) is essential for a complete ABA response under drought conditions, thereby ensuring robust signaling [22,24]. Activated SnRK2s also phosphorylate members of the ABRE-binding protein/factor (ABF/AREB) family of transcription factors. These factors drive the expression of a wide range of ABA-responsive genes that contain ABA-responsive elements (ABREs) in their promoters. The products of these genes often function in osmotic adjustment, stomatal regulation, and cellular protection, thereby substantially improving drought tolerance [25,26,27]. In parallel, drought induces an ABA-independent signaling pathway in which AP2/ERF DREB (dehydration-responsive element-binding protein) factors are robustly upregulated. These DREB factors frequently cooperate with additional transcriptional regulators, predominantly from the NAC and WRKY families, to activate drought-protective gene expression through distinct *cis*-elements [27]. Soybean transcriptome analyses under drought conditions revealed a significant increase in *PYR*/*PYL* and other ABA pathway genes and the activation of DREB and related transcription factors. This finding illustrates that both the ABA-dependent and ABA-independent response branches are mobilized simultaneously in this species [25]. This dual engagement provides soybeans with a flexible and robust transcriptional framework for coping with complex and fluctuating drought environments. The activation of these pathways results in the upregulation of genes associated with osmotic adjustment, such as those involved in proline, raffinose, and galactinol biosynthesis. It also leads to stomatal closure, the detoxification of reactive oxygen species, the regulation of root architecture, and growth arrest [22,28]. These processes collectively promote drought tolerance. For example, ABA-induced regulation can reduce xylem diameter to increase hydraulic efficiency and adjust the root-to-shoot ratio, enabling substantial osmotic protection under water deficit in soybean [28,29]. Genome-wide analyses of *G. max* revealed that ABA not only induces but also suppresses a broad spectrum of genes, thereby modulating cell growth, photosynthetic efficiency, and stomatal conductivity [28]. ABA responsiveness differs across soybean cultivars, and this difference is due to genotype-dependent variability. Improved drought survival has been linked to the overexpression of ABA-responsive genes, such as *GmABF3*. In contrast, drought tolerance can be suppressed by negative regulators, such as *GmPRR3b*, through the downregulation of ABA-inducible transcription factors [25,26].

Drought-responsive microRNAs (miRNAs) modulate growth-defense trade-offs and hydraulic adjustment. The conserved miR169 family represses the cooperation of NF-YA subunits with NF-YB/C to bind CCAAT boxes. This axis regulates stomatal conductance and metabolic reprogramming in legumes, including soybeans, in response to dehydration [29,30,31]. Furthermore, miR393 targets the F-box auxin receptors TIR1/AFB, dampening auxin signaling. It helps restrict growth and stabilize root architecture. These methods are better suited for limited water. Finally, miR396 acts through GRF TFs. It curtails cell proliferation. However, it can be redeployed to improve stress resilience. The management of dosage and spatial patterns is also key in soybean [32,33,34]. These miRNAs couple hormone fluxes with morphophysiological adjustments, especially in roots (e.g., branching angles and lateral density) and reproductive organs, aligning sink strength with supply under water deficit.

At the chromatin level, cytosine methylation adjusts the states of promoters and transposons during stress and recovery. Studies of plants place RNA-directed DNA methylation (RdDM) at the core of CHH methylation dynamics. Soybeans possess canonical RdDM components and salt- and stress-responsive remodelers [35]. While most evidence of methylome priming is plant-wide, soybeans exhibit dynamic, TE-focused CHH methylation during seed development, which is consistent with the idea that transposon neighborhoods are sensitive regulatory compartments [36]. ABA/DREB transcription, miRNA circuits (e.g., gma-miR169, gma-miR393, and gma-miR396), and DNA methylation shape the amplitude and persistence of drought responses in soybean.

### 2.2. Heat: HSF/HSP Network and Chromatin Variants

The ability of soybean to tolerate heat stress is driven by a complex molecular network, with heat shock transcription factors (HSFs) and heat shock proteins (HSPs) playing a primary role. This network also involves changes in the structure of chromosomes and posttranscriptional redox regulation [9,37]. HSFs act as primary regulators of the heat stress response, activating highly conserved pathways that strengthen cellular proteostasis under thermal stress. Key members of the HSF family, such as *GmHsfA1* and *GmHsf*-*34*, are well characterized for their central roles in conferring thermotolerance. Specifically, they bind to HSEs in the promoters of target genes, and their activation leads to the induction of a broad range of HSP families, including HSP70, HSP90, HSP101, and several small HSPs (sHSPs) [38,39]. Studies that focused on the function of *GmHsfA1* revealed that increasing the levels of this gene’s product led to an increase in the levels of GmHsp70. This protein helps plants withstand high temperatures by promoting efficient protein folding, preventing aggregation of denatured polypeptides, and maintaining cellular homeostasis during heat episodes. Similarly, the nucleus-localized GmHsf-34 protein drives improved tolerance to drought and heat by activating HSPs and stress-responsive gene networks [38,39]. Proteomic and transcriptomic analyses of various soybean cultivars revealed that heat-tolerant cultivars exhibit robust, early, and sustained induction of HSPs in response to increased temperature. This expression pattern was strongly correlated with the retention of photosynthetic function and reproductive viability under heat stress, which are vital determinants of yield stability during environmental challenges. The increased synthesis and chaperone activity of HSP70, HSP90, and sHSPs mitigate the harmful effects of protein misfolding and aggregation, which exacerbates thermal stress. Comprehensive molecular characterization also revealed that the early activation of HSFs and their target HSPs, along with additional factors such as DREBs and late embryogenesis abundant (LEA) proteins, contributes to the overall resilience of soybean plants exposed to abiotic stress, highlighting the significance of these factors as strategic targets for molecular breeding and genetic engineering [9,38,39].

Soybean plants have a regulatory layer at the chromatin level that is very sophisticated and further defines their response to heat stress. Recent epigenomic research across various species, particularly plants, has shown that high temperatures cause the specific removal of a histone variant, H2A.Z, from nucleosomes near the start regions of thermoresponsive genes. This substantially enhances chromatin accessibility, facilitating the recruitment of master transcriptional regulators, including heat shock factor A1a (HSFA1a) and phytochrome-interacting factors (PIFs), and enables a rapid transcriptional response to thermal stress [40,41]. Although heat-induced H2A.Z eviction has not yet been mapped directly in soybean, leaf histone-profiling datasets that include H2A.Z, together with heat-induced shifts in chromatin accessibility, support the possibility that a similar thermosensory mechanism operates in *G. max*. Here, we therefore discuss H2A.Z dynamics as a conserved regulatory model for soybean, inferred from shared upstream regulators and the presence of promoter-proximal H2A.Z-marked nucleosomes in soybean chromatin profiles. This process is subject to active regulation by ATP-dependent chromatin remodeling complexes, including INO80, which modulate the dynamics of nucleosome remodeling. Selective removal of H2A.Z at elevated temperatures results in permissive promoter environments, affording transcriptional activators unobstructed access to DNA and, consequently, enhancing the inducibility and amplitude of heat stress gene expression. The SWR1 complex, which contains actin-related protein 6 (ARP6), is pivotal in H2A.Z deposition under basal conditions. However, its removal is facilitated by epigenetic modifiers, including histone deacetylase 9 (HDA9), which promote deacetylation and eviction during heat stress [40,42,43]. Although a substantial portion of this framework has been elucidated in *Arabidopsis* and other model species, emerging transcriptomic and epigenomic datasets from soybean reveal analogous processes. Substantial shifts in both histone modification patterns and chromatin accessibility in soybean are induced by heat stress, further substantiating the universal nature of chromatin-level thermal perception and regulation.

Another vital layer of heat tolerance is regulated by miRNA-mediated control of redox homeostasis. Conserved miR398 is upregulated during heat stress and targets the copper/zinc superoxide dismutase genes *CSD1* and *CSD2* and their cochaperone *CCS*. Through this targeting, miR398 fine-tunes the abundance of superoxide dismutases and modulates intracellular reactive oxygen species (ROS) levels. ROS act as stress signals and, in excess, as sources of oxidative damage; thus, redox adjustment is crucial. Studies in soybean confirmed that gma-miR398 modifies *superoxide dismutase* (*GmSOD*) transcript levels under heat stress, linking posttranscriptional control to increased ROS detoxification and overall thermotolerance [44,45,46]. Integrating *GmHSF*/*GmHSP* transcriptional programs, temperature-sensitive chromatin dynamics governed by H2A.Z, and gma-miR398-based redox tuning results in the formation of a tightly connected regulatory network. These layers work together to maintain the function of flowers and pollen and develop seeds, thereby influencing the reproductive success and yield stability of crops during episodic heat events.

### 2.3. Salinity: SOS Pathway, Osmoprotection, and miRNA-Guided Nutrient Homeostasis

Salinity represents a persistent environmental challenge for soybeans, impairing osmoregulation, ion homeostasis, and metabolic integrity. The response of crops to excess sodium and chloride ions involves tightly regulated coordination of signaling, metabolic, and epigenomic pathways.

A central element of the plant response to salt stress is the *salt-overly sensitive* (*SOS*) signaling pathway involving *Calcineurin B-like Protein 4* (*CBL4/SOS3*)–*CBL-Interacting Protein Kinase 24* (*CIPK24/SOS2*)–*SOS1*. This pathway is responsible for the extrusion of Na^+^ and the maintenance of a balanced relationship between K^+^ and Na^+^ in the cytosol. Other transporters (such as High-affinity K^+^ Transporter (HKT) retrieval, Sodium/Hydrogen Antiporter (NHX) vacuolar sequestration, and AKT/KAT K^+^ channels) and compatible solutes work together to ensure the stability of ion homeostasis and water relationships [47].

Conserved nutrient-responsive miRNAs integrate sulfur and phosphate metabolism with salinity adaptation. miR395 regulates sulfate assimilation and transport (*APS*, *SULTR*), thereby shaping the glutathione-dependent antioxidant capacity relevant to salinity [48,49]. The miR399–*Phosphate 2*(*PHO2*) module adjusts phosphate homeostasis, and molecular manipulation of this axis alters salt responses in *Arabidopsis*. This axis is widely discussed in plant nutrient–stress frameworks and has implications for crops, including soybean [50,51]. In addition to nutrient coupling, growth–stress trade-offs are modulated by the targeting of GRF transcription factors by gma-miR396: in soybean, the effects of gene editing and the overexpression of gma-miR396a on branching, yield, and salinity tolerance demonstrate opportunities for improvement that depend on dosage and stage [52]. Redox regulation is connected to the miR398 family. Analysis of plants and soybeans revealed that it targets *CSD1*/*CSD2* and *CCS*. These findings indicate that ROS signaling is linked to antioxidant protection during salt exposure [53].

Epigenomic remodeling provides a stable framework for these RNA- and transporter-centered layers. High salinity often increases CHH methylation in transposable element (TE)-rich regions via RdDM. This pattern has been observed in crop methylomes under salt stress and is thought to buffer transcription adjacent to TEs [54,55]. Soybean encodes canonical RdDM components and CLASSY family remodelers that route RNA Polimerase IV (Pol IV) activity toward 24-nt small interfering RNA (siRNA) production, and these genes exhibit stress-induced expression, indicating the capacity to redirect methylation during stress [35]. Independently, soybean seeds clearly enriched CHH over TEs during development. These findings indicate that TE-focused methylation dynamics are present in this genome. This finding also supports the idea that TE neighborhoods are regulatory compartments that respond to salinity and likely participate in buffering it [36].

Together, these findings suggest that soybean salt tolerance may emerge from the integration of SOS-anchored ion flux control, HKT/NHX modules, and osmotic adjusters; nutrient-linked miRNA circuits (e.g., gma-miR395 and gma-miR399) that coordinate sulfur and phosphate economies with antioxidant and membrane maintenance; growth and allocation tuning via gma-miRNA396-*GRF*; redox set-point control via gma-miRNA398-*CSDs*; and RdDM-dependent CHH reinforcement at TE neighborhoods that stabilize gene expression under ionic and oxidative stress. These elements have been validated in soybeans and are strongly supported by plant-wide evidence with crop examples. They provide concrete levers for breeding. In practical terms, transporter haplotypes and their promoters, miRNAs with clear target–phenotype links (e.g., gma–miR396a), and RdDM-associated remodelers (CLASSY) are high-priority targets for developing markers, making genomic predictions, and making precise cis-regulatory edits [35,47,52].

### 2.4. Integrated and Distinct Regulatory Modules: Stress Core and Pathway Crosstalk

In plants, drought and heat trigger rapid Ca^2+^ signatures and ROS transients that activate kinase cascades and redox sensors. These cascades converge on overlapping transcription factor (TF) families, including the ABA-responsive bZIPs (ABF/AREB), AP2/ERF (DREB), NAC, and HSF families. Comparative analyses detailing single and combined drought and heat exposures highlight these shared modules and how they are coordinated across tissues and stages [22,56]. At the chromatin layer, high temperatures promote the removal of H2A.Z from promoter-proximal nucleosomes. This process is sometimes coordinated with the deposition of H3.3 and increased engagement of RNA polymerase II at thermoresponsive loci. These events facilitate the rapid activation of transcription and involve remodelers, such as INO80, and regulators, such as Phytochrome Interacting Factor 4 (PIF4)/Heat Shock Factor A1 (HSFA1a) [40,57]. Small RNA hubs connect to this core. The miR169-NF-YA axis modulates growth and metabolism under drought conditions, a function that has been tested in recent studies. Moreover, miR398 targets *CSD1*/*CSD2* and *CCS* to regulate redox reactions during heat and drought. Studies on soybeans have confirmed the targeting of *GmSOD* [44,46,58,59]. When considered as a whole, a drought–heat module that links early ionic/redox cues to TF programs and promotes chromatin dynamics is supported by these layers. Salinity adds an ion transport dimension to the osmotic component it shares with drought. In soybean and related crops, the SOS pathway (Gm*CBL4*/*GmSOS3–GmCIPK24*/*GmSOS2*–*GmSOS1*) promotes the extrusion of sodium (Na^+^) ions. It works with the HKT pathway (xylem retrieval) and the NHX pathway (vacuolar sequestration) to maintain the balance of potassium (K^+^) and sodium (Na^+^) ions in the cytosol. Moreover, compatible solute and aquaporin regulation stabilize water relations. Soybean-focused reviews map these pathways to practical breeding targets [49]. Salinity also recruits nutrient-linked miRNAs. For example, gma-miR395 adjusts sulfate assimilation and transport (*sulfate transporters GmSULTRs*), which are relevant to glutathione-based antioxidant capacity. Additionally, gma-miR399-*GmPHO2* regulates phosphate allocation, which has consequences for energy and membrane remodeling under salt stress [48,50,60]. In soybean, the effect of the gma-miR396 dosage on GRF transcription factors has been functionally connected to salt responses and growth–yield outcomes, adding a development-allocation lever to the salinity toolkit [54].

CHH methylation gain over TE-rich neighborhoods is a recurring epigenomic signature of salt exposure. It is mediated by RdDM and is inferred to suppress TE activity and stabilize nearby gene expression under ionic and oxidative stress. Recent reviews and crop methylome studies document this pattern, with additional evidence that stress-responsive RdDM components and remodelers are engaged during salt responses [54,61]. The soybean genome encodes canonical RdDM machinery, and its seeds display dynamic TE-focused CHH methylation during development, which is consistent with a regulatory “buffer” surrounding stress-responsive genes that can be recruited during salinity episodes [54].

ABA accumulates under drought, heat, and salinity conditions and coordinates stomatal closure, osmotic adjustment, and broad transcriptional reprogramming; it also interacts with other hormones to shape outcomes under combined stress [62]. Auxin sensitivity is downregulated through miR393-mediated repression of TIR1/AFB receptors, a module linked to drought and salinity responses that reallocates resources from growth to survival [63,64]. Brassinosteroids (BRs) contribute to thermotolerance and oxidative protection, increasing antioxidant capacity and, in some contexts, synergizing with ABA to support photosynthesis and reproductive performance under stress [65,66]. These hormone nodes operate within, and feed back on, chromatin states at target promoters, integrating with H2A.Z/H3.3 dynamics during heat and TE-adjacent methylation during salinity. ‘These H2A.Z/H3.3 dynamics have been characterized in *Arabidopsis* and related models, and are proposed here as a conserved chromatin module that is likely to function at thermoresponsive soybean loci.’

## 3. Epigenome and ncRNA Maps in Soybean: State of Knowledge

In recent years, epigenome and non-coding RNA (ncRNA) mapping in soybean has expanded rapidly through next-generation sequencing and integrative omics strategies. These strategies have provided unprecedented insight into the molecular layers underlying adaptation, development, and stress responses. DNA methylation signatures, histone modifications, and the actions of non-coding RNAs have emerged as key factors in shaping gene expression patterns relevant to normal growth and environmental plasticity. Recent studies have highlighted the dynamic interplay between DNA methylation, chromatin accessibility, and diverse regulatory RNAs and underscore the central importance of these factors for the stability and fine-tuning of the soybean transcriptome during physiological and stress-induced transitions.

### 3.1. Methylomics of Soybean

Cytosine methylation is partitioned into three sequence contexts: CG, CHG, and CHH (where H = A, C, or T). Analysis across tissues—roots, stems, leaves, and cotyledons—has consistently demonstrated that CG methylation predominates and is most stable genome-wide, followed by CHG. In contrast, CHH methylation is rarer and more dynamic. In soybean cotyledons, CG methylation typically ranges from 65 to 75%, CHG methylation from 40 to 50%, and CHH methylation from 4 to 6% [67,68,69]. Base-resolved soybean methylomes present high mCG values in the gene bodies of broadly expressed loci, strong mCHG values across heterochromatin and pericentromeres, and mCHH islands concentrated in TE neighborhoods and TE–gene boundaries. WGBS has documented these features across tissues and accessions [67,70]. During seed development, the CHH of soybeans progressively increases over TEs and TE-proximal regions, which is consistent with active RdDM during embryogenesis [36,71]. Functional genetics now connects this TE-centered methylation to yield components: CRISPR editing of the seed-expressed DNA demethylase *GmDMEa* decreased methylation at AT-rich TEs and reduced seed size, establishing a direct link between TE-focused methylation and an agronomic trait in soybean [36]. Furthermore, field studies have indicated that CG/CHG/CHH patterns vary among cultivars in response to environmental conditions, including temperature-related shifts in methylation and coordinated changes in chromatin-related transcripts, highlighting the adaptability of the soybean methylome in natural environments and implying that epigenetic variation plays a role in G × E interactions [72]. Across a range of experimental treatments, soybean frequently presents many locally differentially methylated regions (DMRs) with only a negligible shift in genome-wide averages, emphasizing the necessity for locus-level analyses focusing on TE borders and stress-responsive promoters [73]. Together, these soybean-specific datasets delineate an epigenomic landscape in which TE-rich regions act as dynamic hubs of CHH methylation during seed development and are responsive nodes under environmental fluctuations, as documented in seasonal field methylomes [74]. Moreover, functional genetics has demonstrated that the seed-expressed DNA demethylase *GmDMEa* can decisively influence seed size by modulating methylation at AT-rich, TE-adjacent regions [36].

Across stresses, soybean frequently presents numerous local DMRs with only modest changes in genome-wide averages, emphasizing the need for locus-level analyses at TE borders and stress-responsive promoters [73]. Under salinity, the direction of methylation change is tissue- and protocol-dependent: in seedling leaves subjected to salt stress priming, whole-genome assays reported global hypomethylation, whereas functional soybean studies also documented local promoter hypomethylation that facilitates the induction of salt tolerance regulators such as *GmMYB84* [47,74]. The soybean genome encodes canonical RdDM machinery, including CLSY remodelers that guide Pol IV activity, supporting the capacity for CHH regulation in TE neighborhoods in this species [35]. In recovery settings, many stress-induced changes revert toward baseline levels. However, a subset of these changes persists for a short time, which is consistent with the idea of short-term memory. Experiments tracking the poststress recovery of soybean and seasonal field methylomes both support the idea of an environmentally responsive and partly reversible methylome [75].

Soybean encodes the full complement of RdDM core components—Pol IV/Pol V largest subunits (*GmNRPD1, GmNRPE1*) *with GmNRPD2/GmNRPE2*—along with the *Dicer*-*like* (*GmDCL*), *RNA*-*dependent RNA polymerase* (*GmRDR*), and *Argonaute* (*GmAGO*) gene families, which generate and deploy 24-nt siRNAs for de novo cytosine methylation [76]. Soybean-focused work further identifies and annotates *GmCLSY* remodelers that target Pol IV to defined loci and reports stress-linked expression, supporting locus-specific RdDM activity in this species [35]. Field whole genome bisulfite sequencing (WGBS) indicates seasonal CG/CHG/CHH remodeling and explicitly examines 24-nt siRNA distributions around active versus repressed TEs, which is consistent with RdDM engagement in natural environments [72]. Developmental methylomes show CHH accumulation over TEs during seed ontogeny, and editing the seed demethylase *GmDMEa* alters methylation at AT-rich TEs and reduces seed size, directly connecting TE-adjacent methylation to phenotype [36,71]. In contrast, salt treatments of soybean leaves have produced priming-associated hypomethylation and gene-specific promoter demethylation (e.g., *GmMYB84*), indicating that the direction of methylome changes under salinity is tissue- and protocol-dependent rather than uniformly TE-CHH reinforcing [77].

### 3.2. Chromatin and Histone Modifications

In soybean roots exposed to salinity, genome-wide ChIP-seq for H3K27me3 revealed a dynamic reconfiguration of Polycomb-associated chromatin aligned with transcriptional responses [78]. The genes whose expression was induced by salt tended to exhibit a local reduction in H3K27me3 near promoters or first exons, whereas the genes whose expression was downregulated under stress presented deposition or expansion of H3K27me3 over their regulatory regions; RNA-seq integration supported a negative correlation between H3K27me3 and transcript accumulation at the population level. These results establish H3K27me3 as a responsive layer in soybean and demonstrate that chromatin-based repression balances growth and defense under ionic stress [78]. In addition to these salt-focused studies, soybean datasets that couple TF occupancy with histone marks refine our understanding of promoter and enhancer states [79]. In a genome-wide analysis centered on the circadian factor *GmLUX*, de novo ChIP-seq for H3K9ac, H3K27ac, and H3K4me1, together with downloaded tracks for H3K27me3, H3K4me3, H4K12ac, and H3K14ac and matched ATAC-seq, revealed that *GmLUX* peaks reside within enhancer-like or promoter-proximal regions imprinted by active marks (H3K9ac/H3K27ac/H3K4me1) and flanked by H3K4me3 at transcription start sites (TSSs). H3K27me3 distinguished repressed target subsets. Although designed around circadian regulation, these maps provide soybean-native references for interpreting chromatin states in stress TF families (e.g., *GmDREB/GmAP2, GmNAC, GmHSF*) with similar promoter architectures [79]. Other evidence shows that histone marks are found near RNA processing features in soybean leaves [80]. A reanalysis of leaf ChIP-seq profiles for H3K27me3, H3K4me1, H3K4me3, H3K36me3, H3K56ac, and H2A.Z revealed correlations between mark distributions and cotranscriptional splicing efficiency. This finding reinforces the idea that the histone landscapes of soybeans, as captured by ChIP-seq, reflect functional layers of gene regulation beyond transcript abundance [80]. A multitissue ATAC-seq atlas delineated accessible chromatin regions (ACRs) across six soybean tissues and revealed that ACRs constitute ~3.3% of the genome [81]. ACRs are enriched near transcription start sites and distal regulatory elements of expressed genes, colocalize with TF binding (via available ChIP-seq), and display tissue specificity that predicts expression divergence among paralogs from soybean whole-genome duplication. ACRs revealed the overrepresentation of *GmDREB, GmNAC*, and *GmHSF* motifs in motif analyses, suggesting that stress–response circuits are embedded within the accessible cistrome. These data suggest that many stress-inducible promoters are preaccessible or poised and that accessibility is a gateway for TF occupancy and rapid induction [81,82]. Mapping ACRs also informs enhancer logic. The co-occurrence of ATAC peaks with H3K27ac and H3K4me1 around TF binding provides enhancer annotations in soybean. In this genome, distal regulation has historically been more complex to assign due to repeats and polyploidy. Because drought and heat often rely on rapid deployment of TFs to preexisting sites, integrating ATAC time-courses during stress–recovery with these baseline maps should reveal whether soybean relies on opening new sites de novo or modulating nucleosome positioning at premarked elements [78,83].

CUT&Tag experiments in soybean have begun to connect one-dimensional chromatin states to three-dimensional architecture. Under light cues, soybean chromatin forms long-range loops, whose anchors are significantly enriched in H3K27me3, which is consistent with polycomb-associated chromatin participating in higher-order organization. This study leverages chromatin-modification CUT&Tag and 3D genomics to argue that environmental signals reorganize loop landscapes while linked to repressive histone environments. Although this work focused on light rather than drought/heat/salt, the demonstrated feasibility of CUT&Tag for soybean histone marks paves the way for stress-condition profiling, particularly in low-input tissues such as meristems or developing pods [84]. Hi-C maps across cultivated and wild soybean further indicate that repressive marks such as H3K27me3 are associated with domains and long-range interactions, suggesting that Polycomb-rich regions act as hubs in 3D space. Hi-C and histone-mark mapping convergence support a model in which stress-regulated promoters operate within a preorganized chromatin scaffold that may constrain or facilitate their responsiveness [85].

Generally, in plants, the histone variant H2A.Z is involved in thermal sensing to modulate promoter-proximal nucleosomes; warmer temperatures reduce H2A.Z occupancy, which eases the activation of thermoresponsive genes. Although causal eviction studies have focused primarily on *Arabidopsis* and other models, this regulatory logic likely extends to soybean, as it encodes orthologs of master heat regulators, such as *HSFA1*. MAPK signaling, *PIF4*, and H2A.Z deposition was interconnected across all the plants [86]. This finding suggests that there is an upstream entry point that is likely conserved across eudicots. Soybean leaf histone datasets that include H2A.Z confirmed the presence of this variant in many genes; these tracks have been used to interpret splicing-related chromatin features, indicating that the data quality is sufficient for locus-level analyses. Thus, promoter H2A.Z dynamics are plausible at HSF targets and heat shock loci in soybean, meriting direct CUT&Tag/ChIP-seq under temperature shifts [80]. To date, however, heat-induced H2A.Z eviction has not been experimentally resolved in *G. max*, so this should be viewed as a hypothesis grounded in conservation rather than a demonstrated soybean mechanism.

### 3.3. Regulatory Non-Coding RNAs in Soybean

The recent expansion of high-throughput sequencing technologies, such as small RNA sequencing (sRNA-seq), degradome/PARE, and direct RNA-seq (dRNA-seq), has enabled comprehensive profiling of ncRNAs and their regulatory networks in soybean. These transcriptomic and degradome datasets form the basis for understanding multilayered posttranscriptional regulation, particularly in response to drought, heat, and salinity, across distinct tissues, including leaves, roots, and nodules.

**Figure 2:**
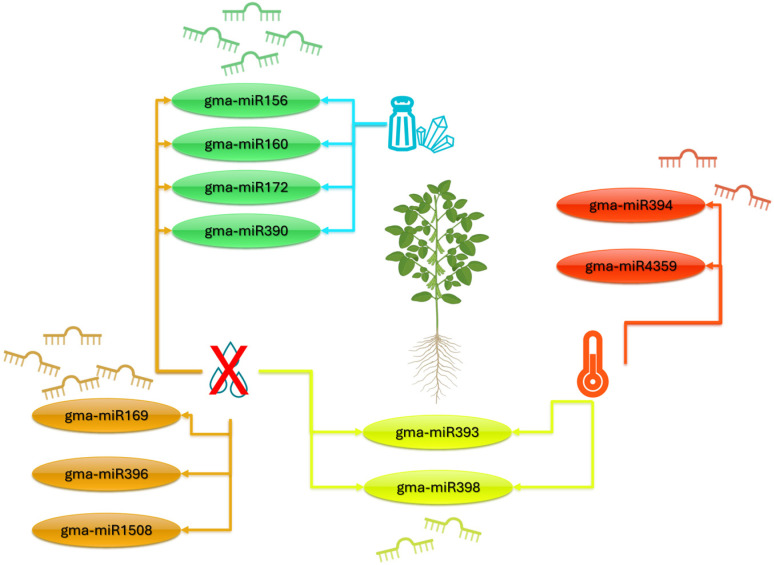
Representative microRNA (miRNA) modules associated with abiotic stress adaptation in soybean. The diagram summarizes literature-reported miRNA families grouped by stress category: salinity, drought, and heat stress.

sRNA-seq has established a comprehensive, tissue-resolved catalog of miRNAs in soybean, revealing condition-specific modulation under drought, heat, and salinity and highlighting recurrent regulatory hubs: gma-miR169, which targets *GmNF*-*YA* subunits and is broadly associated with dehydration responses affecting stomatal behavior and carbon–nitrogen allocation; gma-miR398, which targets *GmCSD1*/*2* and *GmCCS* to tune the reactive oxygen species set-point under drought and heat; gma-miR393, which dampens auxin signaling by regulating *GmTIR1*/*GmAFB* receptors; and gma-miR396, which controls *GmGRF* transcription factors and thereby mediates growth–stress trade-offs (Figure 2). These miRNA cohorts operate across leaves, roots, and nodules—sites where salt and water deficits also perturb symbiosis—underscoring organ specificity and functional breadth [31,87,88,89]. Degradome (PARE) profiling under PEG-simulated drought provides direct evidence of miRNA-guided cleavage for hundreds of targets, including the repression of gma-miR398c coincident with the induction of *GmCSD1*/*2* and *GmCCS*, thereby strengthening causal inference beyond correlative expression [88]. Further connections between miRNA action and phenotype have been revealed by functional genetics: editing or overexpressing Gm-miR396a alters branching and yield components [89]. This improved salinity tolerance, is consistent with a model in which the *GmGRF* dosage integrates growth with ionic stress responses [52]. In addition to these canonical modules, high-confidence inventories consistently document the dynamic regulation of conserved and lineage-enriched families, such as gma-miR1508a, gma-miR156, gma-miR160, gma-miR390, gma-miR394a, gma-miR393a, gma-miR398c, gma-miR172a, gma-miR172c, and gma-miR4359b, induced by both cold and heat across organs and verified to target *pentatricopeptide repeat* (*GmPPR*) genes and *xyloglucan endo*-trans-*glucosylase*/*hydrolases* (*GmXTHs*), linking small-RNA control to photosynthetic performance and cell wall remodeling [30,90,91,92,93,94]. Salinity and drought provoke multilayered responses, both induction and repression, for gma-miR156b, gma-miR160a, gma-miR390, and gma-miR172a, and gain-/loss-of-function studies substantiate their roles in tolerance, root vigor, and shoot growth [87,91,95]. Notably, family members can have different functions. For example, gma-miR172a promotes tolerance to salt and drought, whereas gma-miR172c increases salt sensitivity [95,96]. This emphasizes the need for isoform-level resolution in functional assignments. Among the newer families, gma-miR4359b has emerged via degradome and mutant analyses as a positive regulator of salt survival, improving survival rates and chlorophyll retention under saline conditions [97]. In drought contexts, miR169c, miR393a, and miR398c predominantly act as negative modulators, and loss-of-function alleles confer increased drought tolerance [31,87,88]. In combined-stress paradigms, gma-miR169l-3p, gma-miR5036, gma-miR862a, and gma-miR398a/b respond to multiple stressors, positioning them as integrative hubs with translational value for breeding [88,98,99,100]. A regulatory landscape is delineated by these findings, in which different members of the same family of miRNAs have different functions depending on the situation, and different small-RNA dynamics are connected to photosynthesis, redox homeostasis, hormone signaling, cell wall physiology, and agronomic resilience.

In addition to miRNAs, soybean produces a substantial array of phased small interfering RNAs (phasiRNAs) derived from specific genomic loci (*PHAS* loci), providing a framework for posttranscriptional regulation that intersects with development and abiotic stress. The soybean small-RNA atlas assembled from 69 libraries identified >500 21-nt PHAS loci, most of which are overlapping protein-coding genes, including numerous NB-LRR–like sequences, thereby establishing a reference for phasing registers, triggering miRNAs, and tissue specificity in this crop [101]. Comparative analyses in legumes further indicate that 22-nt miRNAs, together with *Dicer-like 2* (*DCL2*), can initiate 21-nt phasiRNA cascades from target transcripts, a route consistent with soybean genomic evidence for DCL2-dependent small-RNA clusters and with observations that, while many soybean siRNA loci are 24-nt dominant, 21/22-nt clusters frequently exhibit higher abundance [102,103]. Under conditions of prolonged salt stress, soybean roots undergo coordinated miRNA and phasiRNA remodeling. Small RNA and degradome sequencing detect the cleavage of *PHAS* precursors and confirm the presence of target phasiRNAs, thereby linking the miRNA–*PHAS* axis to stress–responsive circuits that extend beyond the scope of canonical defense genes [104]. In simulated PEG drought, integrated analyses combining sRNA-seq, PARE, and transcriptome data capture hundreds of cleaved transcripts and reveal numerous miRNA–mRNA reverse pairs. This phenomenon results in phasiRNA production within broader small RNA networks that recruit antioxidant, hormonal, and metabolic pathways under osmotic challenge [88]. Previous studies on the effects of salt on soybean root tips corroborate these findings by demonstrating global sRNA changes in response to salinity, reinforcing the notion that root sRNA populations, including phasiRNA precursors and products, respond to stress [105]. Developmentally, soybean conforms to the legume pattern in which reproductive phasiRNAs accumulate in anthers (24-nt species in premeiotic stages and 21-nt species later), indicating that the enzymatic components supporting stress-linked phasiRNAs (triggering miRNAs, *RDRs*, *DCLs*, *AGOs*) are also embedded within reproductive programs [106]. Taken together, the soybean datasets support a model in which *PHAS* loci act as regulatory relays that are activated by 22-nt miRNA triggers. These relays then distribute control across gene networks during salinity and osmotic stress. Degradome validation provides direct evidence of precursor cleavage and target engagement. From a practical application perspective, these results suggest the following: editing the trigger site to modulate phasiRNA initiation at selected loci; coupling AGO-IP–sRNA in roots under salt/drought stress to resolve effector complexes; and achieving temporal resolution of PAREs through stress–recovery to distinguish primary phasiRNA-related regulation from the transcriptional consequences that occur downstream. The soybean atlas, the pathways of legume phasiRNA biogenesis, and the stress-induced degradome datasets provide a coherent roadmap for utilizing the miRNA-to-PHAS pathways in breeding programs that target stability in environments with high salinity and a lack of water.

Long non-coding RNAs (lncRNAs) have moved from catalog to function in soybean, with convergent evidence from genome-wide surveys, stress-conditioned transcriptomes, and transgenic validation. Chromosome-scale inventories in *G. max* delineate thousands of lncRNAs with distributions across all 20 chromosomes and characteristic features, lower expression, distinct exon structures, and reduced coding potential relative to mRNAs, thereby providing a reference space in which stress-regulated lncRNAs can be prioritized [106]. On this basis, a transgenic study of soybeans established plant-level causality for lncRNA77580. On this basis, a transgenic study of soybean established plant-level causality for lncRNA77580. The overexpression lines presented increased drought tolerance and increased seed number under water deficit, indicating a positive effect on reproductive performance in dry conditions. However, a clear trade-off emerged under salinity: compared with wild-type plants, lncRNA77580-overexpressing plants were more sensitive to high NaCl at the seedling stage, with reduced growth and survival. Transcriptome profiling revealed that partially nonoverlapping gene sets are shifted by lncRNA77580 depending on whether plants experience drought or salt stress, which is consistent with context-dependent regulation [107]. From a translational perspective, lncRNA77580 exemplifies regulators whose beneficial effects on drought tolerance can be genetically or physiologically coupled to penalties under salinity, highlighting the need to manage such antagonistic correlations when breeding for combined stress tolerance. Earlier studies in soybean focused on lncRNA77580 in the nucleus, showing NaCl-repressed expression in roots, and documenting *cis*-proximal effects on neighboring defense-related genes in engineered hairy roots further support a model in which this lncRNA participates in local chromatin/transcriptional environments while contributing to whole-plant drought performance [18]. On a broader scale, a recent root atlas covering *G. max* and *G. soja* revealed over 1500 and 1400 lncRNAs, respectively, across different root zones and time points, revealing spatially and temporally specific expression patterns [108]. Many lncRNAs that are differentially expressed under abiotic conditions covary with transcription factors that are central to stress responses (e.g., DREB/AREB, NAC, HSF) and with enzymes that govern osmoprotectant homeostasis (e.g., proline and raffinose family pathways), placing lncRNAs at the interface of regulatory and metabolic adaptation. These maps identify tractable candidates for functional assays that link lncRNA dosage to osmolyte accumulation and water-use traits [108]. In addition to stress survival, reviews focusing on soybeans emphasize the roles of lncRNAs in oil traits and seed quality in oil crops, highlighting opportunities for translation that extend from stress tolerance to yield and composition [109]. From a methodological perspective, combining RNA-seq-based discovery and differential expression under drought, heat, and salinity conditions, alongside network integration (e.g., coexpression and WGCNA), has proven effective in identifying candidate lncRNAs. When sRNA crosstalk is suspected, pairing with degradome/PARE and sRNA profiles can clarify whether lncRNAs participate in competing endogenous RNA (ceRNA) relationships or intersect with splicing pathways. Direct RNA sequencing (dRNA-seq) provides complementary information on isoforms and modifications that may affect lncRNA stability and interaction potential. Within this framework, lncRNA77580 is a key reference point, and the growing catalogs of cultivated and wild soybean root samples provide a systematic source of stress-related lncRNAs for targeted editing or expression tuning [18,107,109]. Together, these studies establish the role of lncRNAs as adjustable nodes in soybean stress adaptation, linking transcriptional circuitry and osmoprotectant metabolism to agronomic resilience.

Emerging research utilizing deep-sequencing technologies has revealed an expanding repertoire of circular RNAs (circRNAs) in soybean, indicating their potential involvement in environmental adaptation. CircRNAs, which are covalently closed, exon- or intron-containing RNA molecules, have been systematically cataloged by transcriptome-wide analyses in vegetative and root tissues under distinct abiotic stresses, including low-temperature and low-phosphorus conditions [110]. To date, in soybean, circRNAs have been systematically profiled under two abiotic conditions with strong tissue specificity: low temperature in leaves and low phosphorus in roots [111,112]. More than 370 new circRNA candidates in soybean roots exposed to low-phosphorus (LP) stress have been identified via RNA sequencing (RNA-seq) and specialized algorithms, of which 120 were differentially expressed between stress-sensitive and stress-resistant genotypes [112]. These LP-responsive circRNAs displayed genotype- and condition-specific expression patterns, and network predictions and gene ontology enrichment analyses connected them to pathways controlling phosphate metabolism, stress signaling, and cell wall modification. Nearly 70 differentially expressed circRNAs were computationally predicted to act as miRNA sponges, often binding to stress-associated miRNAs such as gma-miR399, gma-miR319, gma-miR156, and gma-miR159 [112]. This sponging activity is consistent with models from both plants and animals, where circRNAs can modulate posttranscriptional regulatory circuits by sequestering miRNAs from their mRNA targets [113]. In a separate study, circRNAs were identified in soybean leaves subjected to cold stress (low temperature), revealing extensive transcriptomic reprogramming. Leaves exposed to cold presented differential expression of numerous circRNAs compared with the control group, with some also showing co-expression with parent genes involved in adaptive photosynthesis, hormonal interactions, and energy metabolism [111]. The observations revealed two findings: first, that circRNAs affect the expression of genes in the host; second, that they might play a role in regulating genes through interactions with small RNAs. Further functional prediction analyses confirmed the potential coding capacity of several circRNAs, extending their biological significance beyond the traditional role of non-coding RNAs [111]. Despite these catalogs and bioinformatic advances, direct functional investigations of the role of circRNAs in stress adaptation in soybean are limited compared with those of miRNAs or lncRNAs. Nevertheless, the concordant finding of distinct circRNA landscapes across multiple abiotic stresses strengthens the hypothesis that circRNAs play a role in stress reprogramming. The presence of stress-responsive circRNAs, their predicted miRNA binding partners, and their dynamic expression patterns in different organs underscore their likely participation in tissue-specific regulatory networks [112]. Furthermore, advances from broader plant systems indicate that circRNAs can also interact with RNA-binding proteins (RBPs), i.e., a regulatory dimension still largely unexplored in soybean but central for mechanistic dissection in the future [110]. To progress beyond correlation and predictive models, assays linked to the degradome and RBP-centered interactomics are needed to establish the functionality and targets of soybean circRNAs under stress. It is essential to clarify whether observed circRNA responses cascade through modulation of the miRNA network, influence hormone signaling, or integrate with broader transcriptomic and proteomic shifts underlying stress acclimation. This can be achieved via integrative approaches.

### 3.4. Single-Cell and Spatial Perspectives

Breakthroughs in single-cell and spatial transcriptomics have revolutionized our understanding of cell heterogeneity, gene regulatory networks, and adaptive responses in complex plant organs, including soybeans. Technologies such as single-nucleus RNA sequencing (snRNA-seq), single-nucleus ATAC sequencing (snATAC-seq), and multiple spatial transcriptomics methods (including 10x Genomics Visium, Slide-seq, and MERFISH) have enabled us to capture the spatial organization and transcriptional states of individual cells in unprecedented detail in tissues such as seeds and roots [114]. Recent soybean studies have established high-resolution single-nucleus atlases across key organs. A root–nodule atlas resolved 17 cell types, including six nodule-specific identities, and captured transcriptional gradients across infection and fixation zones. The same study provided a functional example of moving from atlas inference to gene validation by demonstrating that *GmFWL3,* a plasma membrane microdomain-associated protein, influences rhizobial infection [115,116]. Independent work integrating single-nucleus and spatial transcriptomics anchored these cell states in situ, revealing a transitional infected subtype and specialized uninfected neighbors that together shape the nodule’s central infection zone [117]. In addition to its role in symbiosis, single-nucleus RNA-seq of cotyledon-stage seeds has revealed multiple seed cell identities, providing a template for investigating how drought, heat, and salinity reprogram storage and protective functions during seed filling [118]. The field has also delivered a spatially resolved multiomic single-cell atlas across ten soybean tissues (~316,000 nuclei), jointly profiling gene expression and chromatin accessibility, thereby enabling analysis of regulatory programs that are shared or organ specific. The preprint and open-access peer-reviewed versions emphasize cross-organ integration and provide a scaffold for projecting stress datasets onto a standard cell-type reference [19]. In parallel, an integrated transcriptomic atlas aggregated bulk RNA-seq data from 314 samples. It links these datasets to single-nucleus and spatial datasets for five organs, facilitating the deconvolution of bulk stress studies and label transfer into spatial data [119]. The availability of snATAC-seq within the multiomic atlas enables the dissection of the *cis*-regulatory logic that underlies stress responses. For each cell type, a simple approach is to measure whether stress TF motifs (e.g., DREB/ABF, NAC, HSF) are found in preaccessible peaks or if accessibility increases during exposure to abiotic stresses, suggesting enhancer activation. In other crops, single-cell datasets under heat conditions revealed cell type-specific heat responses with rapid activation of HSF modules; by analogy, soybean roots and leaves can be profiled at short time scales to determine whether the epidermis, cortex, and stele show distinct heat-inducible accessibility and transcriptional activation and how these patterns resolve upon recovery [120]. As a baseline, tissue-scale ATAC-seq was performed on soybeans to define ACRs across six tissues. It is closely associated with gene expression and supplies coordinates for conserved non-coding elements that can be revisited at single-nucleus resolution in response to stress [81]. In soybean, for salinity, snRNA-seq/snATAC-seq can localize expression and accessibility at ion homeostasis loci, i.e., *GmSOS1*, *GmHKT*, *GmNHX*, and *GmAKT*, across the epidermis, cortex, endodermis, and stele and determine whether specific enhancer–promoter pairs become coaccessible in endodermal barrier cells or the xylem pole pericycle, where Na^+^/K^+^ partitioning is critical. For drought, single-nucleus data can be used to quantify ABA-responsive modules (ABF/AREB, PP2Cs, SnRK2s) in guard cells, mesophyll, bundle sheath, and root endodermis to test whether primed enhancers exist in water-sensing lineages [19]. To study heat, promoter-proximal features associated with thermosensory regulation (e.g., HSF access) can be examined alongside chromatin changes to identify thermoresponsive hubs in developing leaves or root tips. A soybean-wide multiomic reference is available, which allows these analyses to proceed with shared embeddings and consistent cell-type labels. This increases the statistical power for differential accessibility and gene activity scoring [19]. Spatial methods restore the positional context and discriminate microgradients averaged out in dissociated assays. In soybean, integrated single-nucleus and spatial analyses of nodules map infected and uninfected cell states to their anatomical niches, clarifying how transitional infected cells arise and how neighboring uninfected cells contribute to the functional architecture of the infection zone [117]. Plant-focused reviews highlight soybean as an early application of combining snRNA-seq and spatial methods (e.g., Stereo-seq) and discuss how Visium/Visium HD and Slide-seq can capture organ-scale views with varying spatial resolutions [121,122]. These platforms can be used in various ways in drought, heat, and salinity experiments. For example, they can map salt fronts along the longitudinal axis of the root and across radial layers, allowing researchers to identify areas of the endodermis and cortex with increased expression of ion transporters and signaling components. They can also be used to resolve heat microgradients in leaves, e.g., HSF/HSP-enriched rims or vascular-adjacent domains, by contrasting sun-exposed and shaded sectors. Moreover, they enable the tracking of drought-induced heterogeneity, such as suberization fronts in the endodermis, aquaporin redistribution across the cortex and stele, and stomatal modules in leaves. Where MERFISH is available, targeted panels can resolve the subcellular distributions of specific stress transcripts (e.g., aquaporins, HSPs, LEA proteins) in selected regions, complementing whole-transcriptome spatial maps with high sensitivity [121,122]. A pressing objective is to integrate single-nucleus and spatial signals to infer the location of cell states and to predict the expression of transcripts below detection in spatial assays. Methods for joint embedding, label transfer, and imputation can map cell states from snRNA-seq/snATAC-seq onto spatial grids and “fill in the blanks” for weakly captured genes, which is especially useful for rapidly induced TFs or low-abundance transporters under stress. The soybean integrated transcriptomic atlas supports deconvolving bulk stress datasets into cell-type proportions and distinguishes population shifts from per-cell changes [119]. Multiomic modeling that integrates snRNA-seq and snATAC-seq data enables the estimation of gene activity scores at enhancers and promoters, the inference of coaccessibility networks, and the prioritization of *cis*-elements for editing. Researchers can test whether changes in accessibility at enhancer hubs are related to spatial gene induction along stress gradients, which can help identify high-value regulatory elements that structure the stress response in specific areas [19]. Candidate enhancers that regulate stress responses in narrowly defined tissues can be identified via cell type-resolved chromatin maps (e.g., endodermal barriers for salt, guard cells for heat/drought). In contrast, spatial maps can prioritize loci with distinct responses to specific stimuli (e.g., salt entry points or hot leaf margins). *Cis*-regulatory candidates can be tested with promoter/enhancer reporters in soybean hairy roots or transient assays, followed by cis-editing (base/prime editing) to adjust the motif content of DREB/ABF, NAC, HSF, or ion-homeostasis TFs. The root–nodule atlases provide a proof-of-concept workflow, i.e., atlas discovery, marker selection, and functional validation, that can be adapted to abiotic stress modules [116].

## 4. Epigenetic Stress Memory and Inheritance

Epigenetic regulation, which includes DNA methylation, histone modifications, and non-coding RNAs, provides plants with dynamic control over gene expression in response to environmental challenges. This responsiveness enables rapid and reversible acclimation, and in some cases, ‘remembering’ of previous stress exposures—a phenomenon known as ‘epigenetic memory’. In soybean and other legumes, high-resolution mapping of the methylome and chromatin features has revealed how these epigenetic signatures are maintained, reset, or transmitted across cycles of stress and recovery, shaping both immediate adaptability and long-term resilience. Most explicit chromatin memory paradigms have been studied in *Arabidopsis* and cereals. Therefore, we treat these rules as a framework for hypothesis-driven experiments in soybeans rather than as demonstrated mechanisms in *G. max*.

### 4.1. “Priming” in Stress–Recovery Cycles: Lasting Epigenetic Signatures and Secondary Response Timing

In crop plants, priming refers to preparatory exposure that increases the speed and/or magnitude of a subsequent response. A study investigating salt priming in soybeans demonstrated that primed seedlings exhibit global DNA hypomethylation in their leaves, decreased total DNA methyltransferase activity, and altered expression of genes related to methylation. These molecular changes coincide with improved growth and survival during a subsequent salt challenge [77]. The same study also reported changes in histone marks at stress-associated loci during the priming and triggering phases, suggesting that the combined configuration of the methylome and histones contributes to an accelerated secondary response. This soybean-specific evidence places epigenetic remodeling at the core of priming, and importantly, it indicates that significant phenotypic gains can arise without wholesale shifts in genome-wide methylation averages [77]. Across plant systems, cold and heat memory studies converge on two histone features that align with enhanced reinduction. First, H3K4me3 tends to accumulate at promoters of genes that are rapidly re-expressed upon a second stimulus, and H3K4me3 can persist through recovery at levels sufficient to facilitate reactivation. Second, H3K27me3 often shows context-dependent relaxation at inducible loci during priming and/or subsequent exposure, with partial restoration after stress subsides [53,123,124]. Together, these signatures map onto the operational definition of memory-shorter latency and higher amplitude in the secondary response. While many explicit time courses have been performed in model species, the underlying chromatin logic is broadly conserved and amenable to CUT&Tag/ChIP-seq time series in soybean across priming, recovery, and triggering [123,124]. The soybean methylome exhibits environmentally responsive plasticity under natural regimes. A seasonal field study tracked 24-nt small RNAs, the canonical initiators of the RdDM pathway, and CHH methylation at neighborhoods flanking active and repressed TEs. The abundance of 24-nt siRNAs and the extent of CHH methylation varied with environmental conditions, which was consistent with the tuning of TE-proximal chromatin via RdDM. Stress-recovery cycles can establish local DMRs near TE borders and stress-responsive promoters, leaving genome-wide methylation relatively stable, as the available data suggest [72]. This behavior, which involves many local changes with minimal global displacement, is a recurrent theme in abiotic stress and pathogen challenge, and soybean WGBS reinforces it under *Phytophthora sansomeana*, where numerous local DMRs are detected without significant movement in global averages [73]. Independent evidence links histone dynamics to abiotic responses in soybean roots. A genome-wide ChIP-seq analysis conducted under saline conditions revealed substantial remodeling of H3K27me3, which aligned with transcriptional reprogramming. Specifically, a portion of stress-inducible loci lost local H3K27me3, whereas modules associated with growth gained this repressive mark. This antagonistic reallocation is consistent with functional prioritization during ionic stress. Although the design did not include a formal priming step, the results clearly demonstrate the existence of a flexible H3K27me3 layer under salt stress, a prerequisite for histone-based memory in soybean [73]. Converging observations of salt-primed hypomethylation, seasonal RdDM/CHH dynamics and salinity-driven H3K27me3 remodeling support a testable model in which soybean priming engages local hypomethylation (often in the context of CHH at TE-proximal regions) and histone adjustments (e.g., retention of H3K4me3 at fast-response promoters and focal relief of H3K27me3), which persist through recovery long enough to facilitate faster reinduction and greater amplitude during secondary exposure [77]. Operationally, the model predicts an enrichment of persistent DMRs at TE borders and at the promoters of ABA- and ROS-linked genes following priming. It also predicts detectable H3K4me3 at these promoters during recovery and a reduction or redistribution of H3K27me3 at stress-favored loci, which is reestablished once conditions normalize [72]. These predictions can be investigated via time-resolved CUT&Tag/ChIP-seq and WGBS/oxBS in conjunction with 24-nt siRNA profiling in matched tissues. A comprehensive priming design includes preconditioning (e.g., sublethal salt exposure for several hours to days), recovery (to allow for partial resetting), and triggering (a second salt exposure), with sampling at multiple time points [125,126]. Chromatin endpoints should include WGBS/bs-amplicon for DMRs, CUT&Tag for H3K4me3/H3K27me3, and ATAC-seq for accessibility. These data should be matched to RNA-seq data to determine their kinetics. In parallel, 24-nt siRNA profiling indicates whether sRNAs linked to RdDM track with CHH changes at TE-proximal sites [71]. The soybean literature already provides anchors for each layer: hypomethylation and DNA methyltransferase (DNMT) shift following salt priming; seasonal CHH/24-nt siRNA plasticity near TEs; and H3K27me3 remodeling in response to salt stress. Taken together, these features define sentinel loci, such as TE-adjacent regions near *SOS*/*HKT*/*NHX* and the promoters of ABA/ROS regulators, where priming marks are most likely to persist and predict the kinetics of the secondary response [69,78,127]. The durability of priming signatures should be considered on two axes: temporal, or how long the marks persist through recovery, and spatial, or in which tissues and cell types the marks are retained. A seasonal methylome study indicated that CHH/RdDM markers are environmentally sensitive yet patterned. This finding suggests that specific TE-proximal neighborhoods may act as dynamic hubs where repeated exposure can temporarily increase small RNA flux and methylation levels [69,77]. Conversely, H3K4me3 at memory promoters may persist for short to intermediate periods, sufficient to bias reinduction. Moreover, H3K27me3 may be locally relaxed and reimposed to mitigate growth penalties [124,127,128]. The soybean salt-priming phenotype demonstrated that such chromatin adjustments can result in measurable agronomic benefits under recurrent stress [77,129]. Synthesis articles and experimental studies across plant systems emphasize that priming is often encoded by coincident chromatin changes, such as H3K4me3 at inducible promoters and tuned polycomb coverage. These changes are sometimes augmented by DNA methylation and small RNAs [123,130,131]. In *Arabidopsis*, heat and cold memory paradigms demonstrate consistent H3K4me3 retention and reversible H3K27me3 changes at memory loci [130,132,133]. Similar designs are now possible for soybeans via CUT&Tag and time-course ChIP-seq. Aligning soybean data (salt priming, RdDM/CHH dynamics, and H3K27me3 remodeling) with these general rules suggests that soybean priming is embedded in a conserved chromatin architecture that integrates methylation, histone marks, and small RNAs to prioritize rapid secondary responses [69,127,133]. Three aspects are critical for causal interpretation. First, the composition of the tissues and cell types must be controlled because bulk profiles can reflect shifts in cell populations [15,85]. Second, accurate sampling timing is essential; persistent H3K4me3 or DMRs may be overlooked without sufficient temporal resolution [71]. Third, coupling orthogonal assays, such as bisulfite amplicons for sentinel DMRs and CUT&Tag for histone marks at the same loci, to transcript kinetics (e.g., time to half-maximal induction, T50) links the chromatin state with response timing [15,85]. When possible, single-nucleus or spatial readouts can identify the location of durable marks in root zones (epidermis, cortex, stele) or leaf neighborhoods (guard cells, mesophyll). This anatomical precision can be used to create priming maps. These strategies will refine the soybean priming model beyond tissue averages and accelerate the identification of cis-regulatory candidates for breeding or editing [78]. One practical outcome of soybean priming studies is the prospect of using diagnostic markers to verify primed status in seed lots or nurseries. For example, targeted bisulfite amplicons at TE-proximal CHH islands that demonstrate consistent hypomethylation following priming, in conjunction with CUT&Tag for H3K4me3 at a few promoters of ABA/ROS genes, could yield a basic signature associated with quicker latency and greater amplitude upon re-exposure. However, this panel should be studied in different genetic backgrounds and environments because soybean methylation is sensitive to the environment but follows patterns, and histone remodeling depends on the context [72].

### 4.2. Transgenerational Inheritance: Persistence of Epigenetic and ncRNA Signatures

The reproduction of angiosperms features a distinctive small RNA economy that intersects with DNA methylation and chromatin states in ways that, under specific conditions, can extend beyond a single life cycle. This framework is centered on 24-nt siRNAs, which are generated by the Pol IV–*RDR2–DCL3* axis and loaded into AGO4/6—these siRNAs then direct DRM2-mediated RdDM at TEs and their flanking regions. During plant reproduction, these siRNAs accumulate in sporophytic and gametophytic tissues, shaping transcriptional landscapes important for genome integrity and seed development [134]. This system is conserved and has been studied in *Arabidopsis*, rice, and other crop species [135,136]. Notably, legumes, including soybeans, encode the complete set of RdDM factors necessary for these processes. Recent studies emphasize that reproductive sRNAs are produced locally and can act non-cell-autonomously to reinforce methylation patterns that influence the next generation [134]. While many of the most resolving experiments have been performed in model species, soybean possesses the canonical RdDM machinery and CLASSY (CLSY) chromatin remodelers that direct Pol IV to specific loci. A 2025 soybean-focused analysis identified new *CLSY1*/*2*-*like* candidates, documented their evolutionary history, and revealed stress-induced expression changes across salt/osmotic conditions [35]. The same study described coregulation with other RdDM components (*AGO4, DCL3, DRM2*) during seed germination under abiotic stress. This finding establishes soybean’s potential for locus-preferential siRNA biogenesis and suggests that reproductive tissues could exhibit CLSY-biased targeting, a prerequisite for patterned RdDM marks with the potential to persist or be rapidly reimposed across generations [35]. Controlled hybridization experiments in maize revealed that divergent parental siRNA pools can induce large sets of differential methylation events (trans-acting epialleles) in F1 progeny. Notably, a subset of these epialleles remained stable through multiple generations of backcrossing and selfing, even when the recurrent parent’s genome became dominant [137]. These findings provide direct evidence that siRNA-mediated DNA methylation can produce transgenerationally inherited methylation states on a large scale in crop species, resulting in changes in gene expression and traits. Although soybeans have not yet been profiled via an identical multigeneration design, the maize results provide a robust template for hypothesis-driven testing in soybeans. Field-based seasonal profiling in soybean revealed that 24-nt siRNA abundance and CHH methylation near both active and repressed TEs vary with the environmental regime. Because 24-nt siRNAs are the canonical initiators of RdDM at CHH sites, these data indicate that soybean methylation landscapes are environment-responsive yet patterned in TE neighborhoods [72]. Such loci are logical candidates for either partial meiotic persistence (via CG/CHG maintenance) or rapid reconstitution in the embryo or early seedling guided by parental/reproductive 24-nt siRNAs, thereby producing intergenerational similarity without necessarily requiring complete stability of every mark through meiosis. Independent of its reproductive biology, soybean exhibits chromatin plasticity at functional loci under abiotic stress. Under salinity, H3K27me3 is remodeled globally in roots in a pattern consistent with functional reprioritization: stress-induced genes lose local H3K27me3, whereas growth-associated modules gain H3K27me3, tightly tracking transcriptional changes [14,78]. Although this study did not implement a formal intergenerational design, it demonstrated that the polycomb layer is dynamic under stress in soybean, fulfilling a key prerequisite for histone-based contributions to memory across cycles of stress and recovery [78,129]. Independent of its reproductive biology, soybean exhibits chromatin plasticity at functional loci under abiotic stress. Research on soybean has demonstrated that heritable epigenetic variation can be captured for breeding through *MSH1* perturbation. In this system, disruption of organellar genome surveillance creates “memory lines” with reproducible methylome reconfiguration and altered development. When these lines are crossed back to the isogenic wild type, progeny families segregate for methylation patterns and agronomic traits despite the near absence of DNA sequence polymorphisms, and epilines show yield and stability gains [138,139,140]. Complementary work in other species has shown that siRNA transmission from *MSH1* rootstocks can drive heritable methylation repatterning and vigor. The loss of siRNA production in the rootstock abolishes these outcomes—implicating RdDM [138,141]. Together, these studies confirm that crop epialleles can be multigenerational and selectable; soybean provides a validated path to exploit epigenetic diversity for improvement. Improved performance upon re-exposure following drought priming has been reported in soybean studies at the whole-plant level, including maintenance of water status and growth under recurrent stress. Other reports (temperature/elevated CO_2_) describe transgenerational effects on seed emergence and vigor. While these experiments did not map chromatin across generations, the phenotypic carry-over is consistent with partial evasion of meiotic reset by selected methylation states and/or reimposition of CHH marks via reproductive siRNAs [125,142]. Research on soybean aligns with the broader plant principle that a fraction of stress-aligned chromatin and small RNA features can influence progeny performance under defined contexts.

### 4.3. Consequences for Breeding: “Primed Genotypes,” Managing Epiallelic Variation, and Agronomic Stability

There are two ways to use epigenetic information to improve soybean plants. The short-term approach builds on somatic priming and uses chromatin signatures as quality metrics for seed or seedling batches intended for repeated stress regimes. The long-term approach leverages heritable epigenetic variation documented in soybeans via the *MSH1* epibreeding system and integrates locus-level epigenomic features into genomic selection pipelines. Together, these methods can convert chromatin states into measurable molecular phenotypes, improving the prediction and management of yield stability under drought, salinity and heat. A salt-priming study in soybean showed that primed seedlings accumulate global DNA hypomethylation in leaves, exhibit a reduction in DNA methyltransferase activity, and display changes in histone marks at stress-relevant loci; these molecular adjustments coincided with improved tolerance during subsequent salt exposure [69,127]. This directly motivates diagnostic panels that quantify priming status via a small set of bisulfite amplicons (sentinel DMRs, preferentially at TE-flanking CHH “islands”) and CUT&Tag readouts for H3K4me3 (poised promoters) and H3K27me3 (Polycomb relief/redeposition) at stress-linked promoters [78,127]. Because priming benefits are context dependent, such panels should be calibrated genotype-by-treatment and validated against response kinetics (e.g., shorter T50 to induction; higher amplitude at the second exposure). The soybean evidence indicates that significant phenotypic gains are compatible with minimal shifts in genome-wide methylation averages, emphasizing the value of locus-level assays over global summaries [69,77,127]. The soybean provides a validated case in which heritable epialleles can be captured and selected. In the *MSH1* system, “memory lines” crossed back to the isogenic wild type produced families that segregated for methylation variants and agronomic traits, with epilines delivering yield and stability gains across environments. While the F2:6 generation attenuated some effects, the platform establishes the feasibility of refreshing epiallelic diversity and embedding epiallelic features into routine selection [138,140]. This soybean result dovetails with cross-crop evidence from maize, where divergent parental siRNA populations induced trans-acting epialleles at thousands of loci, a subset of which persisted across multiple generations—implicating RdDM-mediated sRNA pathways in multigenerational inheritance. Together, these studies justify a breeding track that treats epialleles as partly stable, partly reconfigurable units that can be captured, tested, and rotated within elite pools [138,140]. Seasonal field profiling in soybean revealed environmentally responsive changes in 24-nt siRNA abundance and CHH methylation around active and repressed TEs. Because 24-nt siRNAs initiate RdDM at CHH sites, TE-flanking regions that repeatedly show coordinated siRNA/CHH behavior under natural regimes are strong candidates for adaptive epialleles—loci that either partly persist through meiosis (via CG/CHG maintenance) or are rapidly reimposed in the next generation by reproductive 24-nt siRNAs [127]. These loci are also attractive sentinels for priming diagnostics and Epi-GS: they are structured enough to be predictive across seasons but flexible enough to adjust to local environments [72]. Reviews underscore dense crosstalk between hormones and chromatin layers: ABA signaling frequently aligns with promoter poising and stress memory; auxin interfaces with growth–defense trade-offs and can be reshaped by small RNAs; and brassinosteroids contribute to thermo and oxidative protection with documented network-level interactions. The incorporation of hormone profiles (e.g., ABA/IAA/BR) as covariates with chromatin features may increase the predictability of performance under combined stresses, a scenario common in soybean production [143,144,145]. Finding the right balance is key: on the one hand, there is the need to be stable, which can lead to defensive behaviors and penalties in favorable situations; on the other hand, there is the challenge of adaptability, which can compromise reliability. Advancement should involve stress–cycling trials (priming–recovery–trigger) and multi-environment tests, with longitudinal tracking of sentinel methylation and histone marks. The presence of environment-sensitive yet patterned features in CHH/24-nt siRNAs in soybean suggests that they could be adaptive epialleles [72]. This means that they are responsive but sufficiently stereotyped to be predictable across seasons and locations.

## 5. Perspectives and a Roadmap for Soybean Breeding

Modern soybean breeding targets adaptive, high-yielding cultivars by integrating multilayered regulatory networks, epigenetic diversity, and advanced genomic tools. Recent advances highlight the critical roles of regulatory modules, innovative prebreeding strategies, precision *cis*-editing, and supportive policy frameworks for accelerating soybean improvement.

### 5.1. Priority Regulatory Modules for Validation

Convergent evidence points to several stress hubs in the soybean gma-miR169–*GmNF*-*YA* modules that merit near-term experimental validation regarding their role in shaping drought responses and carbon–nitrogen allocation. These data indicate that specific members of the gma-miR169 family act as negative modulators of dehydration tolerance, while the overexpression of *GmNF*-*YA* alleles can improve performance under salt and drought conditions. These findings position this axis as a tractable, dosage-sensitive node for selection or fine-tuning [146]. Another area of interest is the gma-miR398–*GmCSD* (*GmCSD1*/*2*; *GmCCS*) module, which operates under drought, heat, and salinity conditions. Soybean studies have shown that gma-miR398c is repressed under PEG/drought conditions and that gma-miR398c is functionally linked to antioxidant systems. Recent work has extended the roles of gma-miR398 to include combined stress/virus contexts [88,147]. Another area of interest is the lncRNA “scaffold/switch” candidates, notably lncRNA77580. Transgenic soybean lines demonstrate improved performance under drought and salinity conditions alongside transcriptomic reprogramming. These findings provide evidence of plant-level causality and motivate targeted tests of lncRNAs that interact with ABA, ROS, and osmoprotectant metabolism [107].

### 5.2. Prebreeding Programs: Integrating the Epigenome and ncRNA Profiles

The incorporation of epigenetic and ncRNA data into germplasm characterization has expanded the selection horizon for both *G. max* and wild *G. soja* resources [148,149]. GWASs and dense haplotype mapping have identified elite haplotypes associated with plant height, seed composition, maturity, and stress tolerance [150]. The screening of germplasms for haplotype-epigenetic signatures, such as specific DMRs, active histone marks, and ncRNA expression patterns, enables the selection of superior donor lines for crossbreeding [150,151]. Cataloging regulatory diversity (e.g., gma-miR169–*GmNF*-*YA* or lncRNA-dependent stress signatures) adds precision to germplasm reviews, supports epigenomic selection (Epi-GS), and provides markers for complex traits that are often elusive via sequence-based approaches alone [146,151].

### 5.3. Targeted Cis-Editing: Fine-Tuning Expression Without CDS Modification

Progress in genome editing, particularly via CRISPR/Cas and base-editing technologies, has enabled subtle modulation of gene expression via edits in regulatory regions rather than coding sequences [21]. *Cis*-editing of promoters, enhancers, and ncRNA loci offers advantages for quantitative traits and regulatory constraints, such as adjusting the expression levels of stress tolerance genes without introducing new protein variants [21]. Recent studies on soybean have shown that improving the precision of CRISPR cassette design and promoter editing enhances trait predictability while minimizing pleiotropic effects. This approach is particularly encouraging for breeders seeking specific responses to climate or management pressures [152].

### 5.4. Public–Private Collaboration and Regulatory Frameworks

Efficient deployment requires alignment with science-based regulatory pathways and multisite validation. In the EU, new genomic techniques (NGTs) are the subject of active policy development. The European Food Safety Authority (EFSA) summarizes the scientific aspects of NGTs, and the steps taken by the Council and Parliament in 2025 signal progress toward proportionate oversight for specific edits [153]. In the USA, the SECURE rule (7 CFR part 340) provides exemptions for certain types of editing and a confirmation process to indicate when a plant is not subject to APHIS regulation. Congressional and USDA materials explain how gene-edited plants are managed under the Coordinated Framework. APHIS Agricultural Issues Responses provide case-by-case determinations, including communications concerning soybeans. In Japan, practical guidance has enabled the marketing of edited products, providing a model for the proportionate adoption of risk [154].

## Data Availability

No new data were created or analyzed in this study.
